# Targeting Melanoma-Initiating Cells by Caffeine: In Silico and In Vitro Approaches

**DOI:** 10.3390/molecules26123619

**Published:** 2021-06-13

**Authors:** Claudio Tabolacci, Martina Cordella, Stefania Rossi, Marialaura Bonaccio, Adriana Eramo, Carlo Mischiati, Simone Beninati, Licia Iacoviello, Antonio Facchiano, Francesco Facchiano

**Affiliations:** 1 Department of Oncology and Molecular Medicine, Istituto Superiore di Sanità, Viale Regina Elena 299, 00161 Rome, Italy; martina.cord87@gmail.com (M.C.); stefania.rossi@iss.it (S.R.); adriana.eramo@iss.it (A.E.); francesco.facchiano@iss.it (F.F.); 2 Department of Epidemiology and Prevention, IRCCS Neuromed, 86077 Pozzilli, Italy; marialaura.bonaccio@moli-sani.org (M.B.); licia.iacoviello@moli-sani.org (L.I.); 3 Department of Neuroscience and Rehabilitation, School of Medicine, University of Ferrara, 44121 Ferrara, Italy; msc@unife.it; 4 Department of Biology, University of Rome Tor Vergata, 00133 Rome, Italy; beninati@bio.uniroma2.it; 5 Department of Medicine and Surgery, Research Center in Epidemiology and Preventive Medicine (EPIMED), University of Insubria, 21100 Varese-Como, Italy; 6 Laboratory of Molecular Oncology, IDI-IRCCS, 00167 Rome, Italy; a.facchiano@idi.it

**Keywords:** caffeine, melanoma, melanin, immunomodulatory signals, bioactive compounds

## Abstract

The beneficial effects of coffee on human diseases are well documented, but the molecular mechanisms of its bioactive compounds on cancer are not completely elucidated. This is likely due to the large heterogeneity of coffee preparations and different coffee-based beverages, but also to the choice of experimental models where proliferation, differentiation and immune responses are differently affected. The aim of the present study was to investigate the effects of one of the most interesting bioactive compounds in coffee, i.e., caffeine, using a cellular model of melanoma at a defined differentiation level. A preliminary in silico analysis carried out on public gene-expression databases identified genes potentially involved in caffeine’s effects and suggested some specific molecular targets, including tyrosinase. Proliferation was investigated in vitro on human melanoma initiating cells (MICs) and cytokine expression was measured in conditioned media. Tyrosinase was revealed as a key player in caffeine’s mechanisms of action, suggesting a crucial role in immunomodulation through the reduction in IL-1β, IP-10, MIP-1α, MIP-1β and RANTES secretion onto MICs conditioned media. The potent antiproliferative effects of caffeine on MICs are likely to occur by promoting melanin production and reducing inflammatory signals’ secretion. These data suggest tyrosinase as a key player mediating the effects of caffeine on melanoma.

## 1. Introduction

A large body of evidence points to a positive correlation between habitual coffee consumption and the reduced risk of several diseases including type 2 diabetes and neurological, neurodegenerative and cardiovascular diseases [1,2,3]. In addition, several studies have shown that increased coffee consumption is associated with decreased mortality from different types of cancer including melanoma [4], colorectal [5], prostate [6], lung [7], liver [8], and breast cancer [9]. Coffee’s beneficial effects have been attributed to its bioactive components, such as alkaloids (caffeine) and phenolic acids (chlorogenic and caffeic acids), among others [10]. In fact, caffeine (Caff; 1,3,7-trymethylxanthine) is a purinergic-like molecule found in coffee beans (*Coffea arabica* L. and *Coffea canephora* Pierre ex Froehner), tea leaves (*Camellia sinensis* (L.) Kuntze) and cocoa powder. Caff acts primarily as an adenosine receptor (ADORA) antagonist and as a non-competitive acetylcholinesterase (ACHE) inhibitor [11]. A further interesting pharmacological effect of Caff is related to the competitive antagonism of the phosphodiesterase (PDE) enzyme, which leads to increased intracellular cAMP levels with consequent activation of the AMP-activated protein kinase (AMPK) pathway [12].

Malignant melanoma, one of the most aggressive forms of skin cancer, is an immunogenic tumor characterized by resistance to target therapy [13], great propensity to metastasize, and large heterogeneity [14]. The latter is mainly due to mutations frequently harboring melanoma cells, such as BRAF V600E and others [15]. Several new treatment options have been approved in recent years for melanoma patients, including targeted therapies, immunotherapy, and their combination [16]. However, the clinical efficacy of these treatments is often limited due to the heterogeneity of melanoma [17]. Indeed, the interplay of cancer cells with neighborhood immune cells in the tumor microenvironment (TME) plays a crucial role in melanoma proliferation, progression, and metastasis [18]. Melanoma-initiating cells (MICs), like other cancer stem cells (CSCs), are a cancer sub-population with self-renewal capabilities and are responsible for melanoma progression, relapse, immune evasion, and therapeutic failure [19,20]. An immunosuppressive microenvironment, due to the interactions of CSCs with TME, is known to be crucial for supporting stem cell characteristics and plasticity [21].

We have previously demonstrated that theophylline (1,3-dimethylxanthine) possesses potent antiproliferative, differentiative and immunomodulatory activities against MICs [22]. Therefore, this study aimed to investigate, using additional experimental approaches, the effects of Caff on human primary MICs. As this drug is known to act through several molecular pathways, we started with an in silico screening, to focus in greater detail the effects of Caff on MICs differentiation as well as immunomodulatory signals such as cytokines/chemokines secretion.

## 2. Results

### 2.1. In Silico Analysis of Caffeine Molecular Targets (CMT) in Melanoma

To investigate the effects of Caff on melanoma, we performed an in silico analysis of CMT, which allows the screening of several potential targets. Starting from literature data available on Pubmed, 26 CMT were selected and further analyzed in the GDS1375 dataset (Table 1 and Table S1).

Whereas 14 genes showed no significant differences (Table S1), a total of 12 genes related to CTM (Table 1) were identified as differentially expressed at the mRNA level in melanoma (*n* = 45) and in nevi (*n* = 18) biopsies (*p* < 0.001). For each gene, the calculated fold change in melanoma vs. controls is indicated. ROC curve analyses demonstrated that eight genes (in bold in Table 1) can efficiently discriminate melanoma from nevi samples (Area under the curve, AUC > 0.85 and a *p* ≤ 0.0001). In particular, the AUC was 0.89 for *DNA2* (DNA replication helicase/nuclease 2), 0.87 for *MAPK1* (mitogen-activated protein kinase 1), 0.96 for *PTK2B* (protein tyrosine kinase 2 beta), 0.94 for *ADORA2* (adenosine A2a receptor), 0.95 for *MAOA* (monoamine oxidase A), 0.98 for *PARP1* (poly (ADP-ribose) polymerase (1), 0.94 for *TYR* (tyrosinase), and 0.86 for *HDAC1* (histone deacetylase 1).

**Figure 1:**
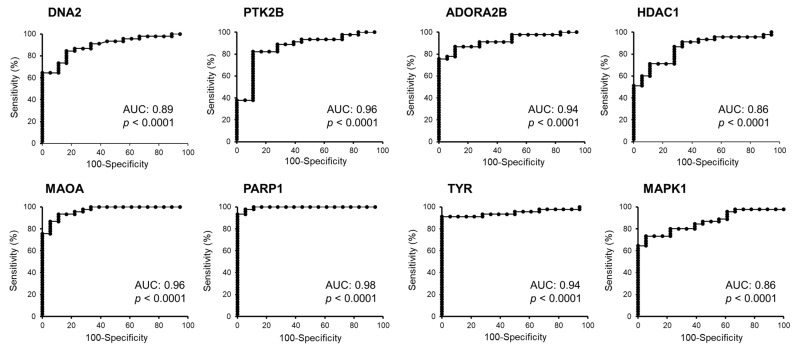
ROC curve analysis to test the validity of expression of selected genes from the GDS1375 dataset (GEO database) to distinguish melanoma from nevi samples (AUC > 0.85 and a *p* ≤ 0.0001). *DNA2* (DNA replication helicase/nuclease 2), *PTK2B* (protein tyrosine kinase 2 beta), *ADORA2B* (adenosine receptor A2b), *HDAC1* (histone deacetylase 1), *MAOA* (monoamine oxidase A), *PARP1* (poly (ADP-ribose) polymerase 1), *TYR* (tyrosinase), *MAPK1* (mitogen-activated protein kinase 1) and their ability to discriminate between melanoma and nevi samples. AUC, area under the curve.

**Figure 2:**
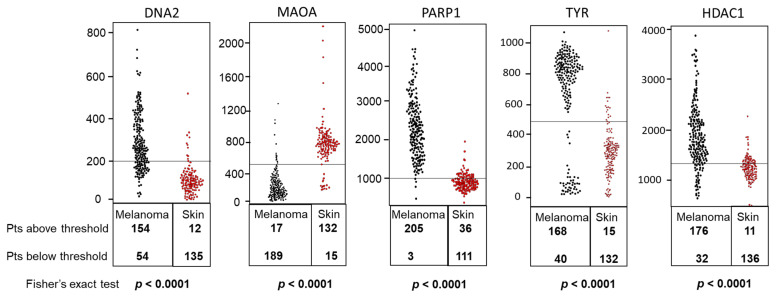
Gene expression study to compare normal (red points) and melanoma (black points) tissues. Selected genes were analyzed using the IST Online database. *DNA2* (DNA replication helicase/nuclease (2), *MAOA* (monoamine oxidase (A), *PARP1* (poly (ADP-ribose) polymerase (1), *TYR* (tyrosinase), and *HDAC1* (histone deacetylase 1) display different expression levels in melanoma vs. healthy skin. The number of samples in each box is indicated. The y-axis indicates the expression levels. Differences in the expression levels of selected genes were evaluated by Fisher exact test (*p* < 0.0001).

**Figure 3:**
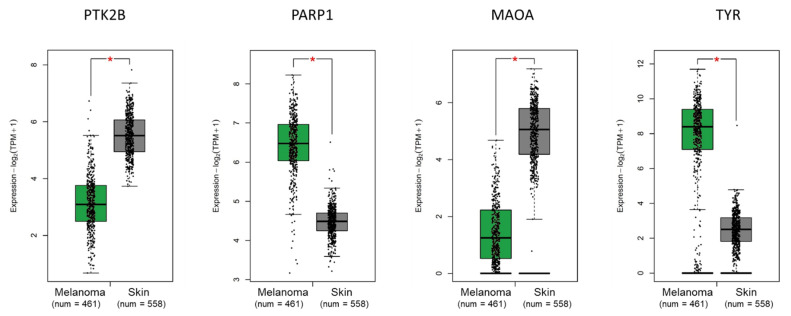
Tissue gene expression according to the GEPIA2 database. *PTK2B* (protein tyrosine kinase 2 beta), *PARP1* (poly (ADP-ribose) polymerase (1), *MAOA* (monoamine oxidase (A), and *TYR* (tyrosinase) show significant (* *p* < 0.01) different expression between melanoma and normal skin. Expression level of selected genes between melanoma and normal tissues was investigated within the TCGA-SKCM (The Cancer Genome Atlas—Skin Cutaneous Melanoma) cohort and the GTEx (Genotype Tissue Expression) GEPIA2 database. The log2FC (fold change) cutoff was 1. A *p*-value cutoff of 0.01, and a jitter size of 4 were used. TPM, transcripts per million.

ROC curves of these selected genes are shown in Figure 1. The sensitivity and specificity values were computed as reported in the Methods section, indicating that these genes, selected among those acting as potential molecular targets of caffeine action, may play a potentially significant role in melanoma growth and/or progression. These eight genes were then validated (see Material and Methods section) by two independent databases, namely IST Online and GEPIA2. Tissue expression was confirmed to be significantly different in cancer vs. control samples for *DNA2*, *MAOA*, *PARP1*, *TYR*, and *HDAC1* in the IST Online database (Figure 2) and for *PTK2B*, *MAOA*, *PARP1*, and *TYR* in the GEPIA2 database (Figure 3). Therefore, thanks to this cross-validation among different databases, *MAOA*, *PARP1* and *TYR* represent the best CMT with significant differential expression in melanoma vs. control biopsies.

**Figure 4:**
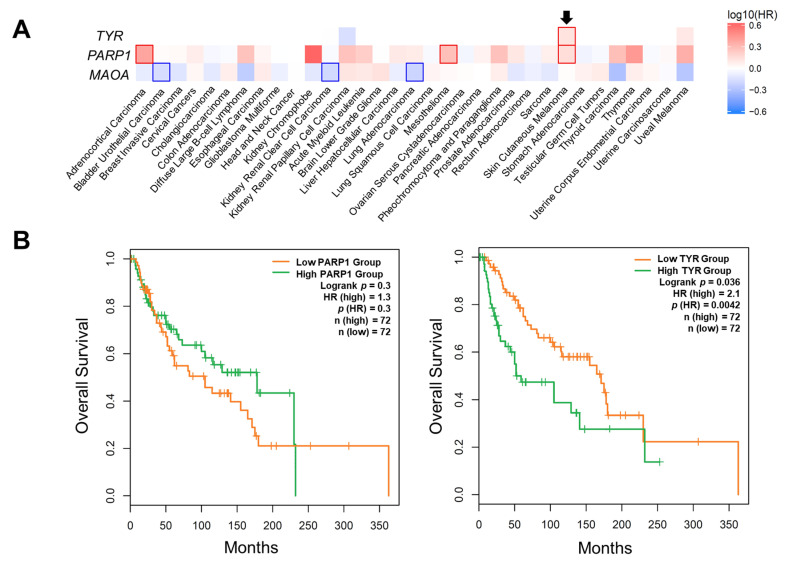
Overall survival analysis with GEPIA2 database. (**A**) Survival heat map of hazard ratio (HR) shows the prognostic impacts of *TYR* (tyrosinase), *PARP1* (poly (ADP-ribose) polymerase 1), and *MAOA* (monoamine oxidase A) on multiple cancer type according to the GEPIA2 database. Median is selected as a threshold for separating high-expression and low-expression cohorts. The red and blue blocks represent higher and lower risks, respectively. The bounding boxes depicted the significant (*p* < 0.05) unfavorable and favorable results, respectively. The black arrow indicates cutaneous melanoma. (**B**) Overall survival (OS) plots showing the prognostic potential of *PARP1* and *TYR* for patients with BRAF-mutated melanoma subtypes. The median overall survival is statistically (*p* (HR) = 0.0042) significant for *TYR*, as indicated in the text in the upper right corner.

Interestingly, TYR (the key enzyme for melanin production) shows specificity of expression in both cutaneous and uveal melanoma (Figure S1). However, to better investigate the role of these CMT, we performed a survival analysis using the GEPIA2 database. As shown in Figure 4A, this analysis of such target genes expressed onto several cancer types reveals that only the increase in PARP1 and TYR expression is related to melanoma severity and survival. More interestingly, we found that low TYR expression displays a significant association with unfavorable prognosis in BRAF-mutated melanoma subtypes (Figure 4B).

### 2.2. Effects of Caffeine on MICs Proliferation, Melanospheres Formation and Melanin Production

**Figure 5:**
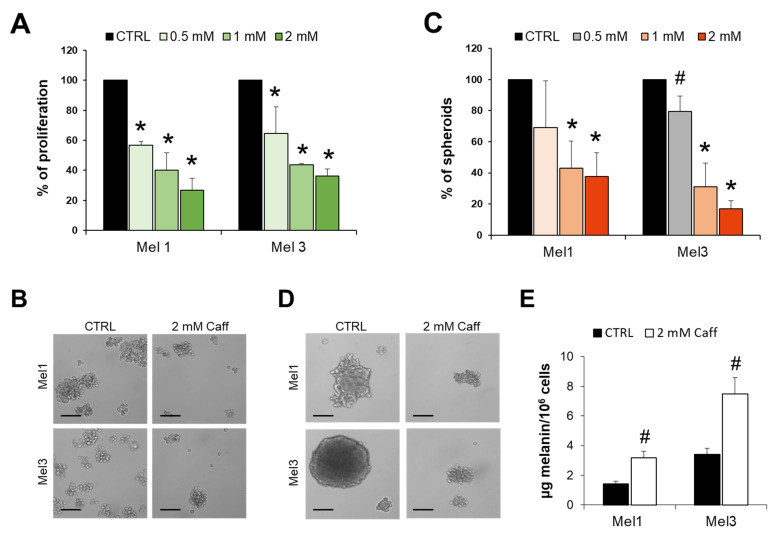
Effects of caffeine (Caff) on MICs. (**A**) Caff (0.5, 1, 2 mM) reduces Mel1 and Mel3 proliferation after 72 h of exposure in a dose-dependent manner. Controls are set at 100%. (**B**) Light microscopical appearance of untreated and 2 mM Caff treated MICs after 72 h of exposure (original magnification: 200×; scale bar: 30 µm). (**C**) Effects of long-term exposure (1 week) to Caff (0.5, 1, 2 mM) on Mel1 and Mel3 spheroids formation. Number and sizes of spheroids were quantified with Image J^®^ (Wayne Rasband, National Institutes of Health, Bethesda, MD, USA). Controls are set at 100%. (**D**) Light microscopical appearance of untreated and 2 mM Caff treated MICs spheroids after 1 week of exposure (original magnification: 200×; scale bar: 30 µm). (**E**) Caff induces melanin production in Mel1 and Mel3 cells. The data are presented as mean ± SD and are representative of three independent experiments (statistical significance: * *p* < 0.01, # *p* < 0.05).

In order to validate the in silico results, we investigated the effects of Caff on in vitro models of BRAF-mutated MICs. Treating Mel1 and Mel3 cells with increased concentrations of Caff significantly reduced cell proliferation in a dose-dependent manner, with a strong and significant reduction in cellular density after 2 mM Caff treatments (Figure 5). The ability of Caff to limit stemness behavior, namely the formation of spheroids (long-term exposure), was directly investigated by measuring the formation of melanospheres. As shown in Figure 5C,D, Caff induces a significant reduction in the size and number of spheroids, which reached 63% and 83% for Mel1 and Mel3, respectively (percentage of controls).

Based on the in silico results described above, the effects of Caff on melanin production in MICs were investigated. It is known that TYR (EC 1.14.18.1) catalyzes the oxidation of tyrosine to l-3,4-dihydroxyphenylalanine (l-DOPA) and represents the rate-limiting enzyme of melanin synthesis [23]. A two-fold increase in melanin production, after 2 mM Caff treatment, was found in both Mel1 and Mel3 cells (Figure 5E), thus confirming the in silico data and supporting the hypothesis that Caff inhibits the growth of BRAF-mutated melanoma cells through a differentiation-inducing pathway. 

**Figure 6:**
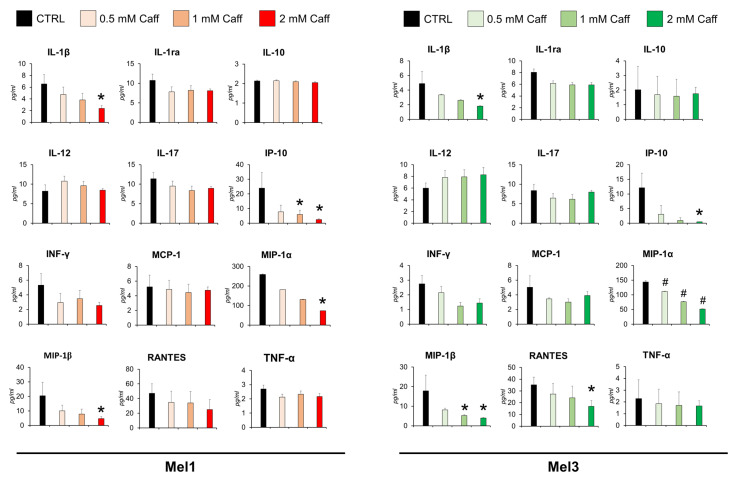
Caffeine (Caff) affects cytokines/chemokines secretion by Mel1 and Mel3 cells. Conditioned media were analyzed using xMAP technology to measure the levels of indicated molecules after 72 h of Caff exposure. This technology, carried out on a X200 Luminex platform (Bio-Rad, Hercules, CA, USA) (see Materials and Methods section), allows the simultaneous measurement of many analytes within the same sample. Data, reported as pigograms/mL, are presented as mean ± SD (statistical significance: * *p* < 0.01, # *p* < 0.05). Analyzed molecules: interferon-inducible protein 10 (IP-10 or *CXCL10*), monocyte chemoattractant protein-1 (MCP-1 or *CCL2*), macrophage inflammatory protein 1-α (MIP-1α or *CCL3*), MIP-1β (*CCL4*), regulated and normal T cell expressed and secreted (RANTES or *CCL5*), interleukin (IL)-1β (IL-1β or *IL1B*), IL-10, IL-12, IL-17, interleukin-1 receptor antagonist (IL-1ra), tumor necrosis factor-alpha (TNF-α), and interferon-γ (IFN-γ).

It is well known that Caff, as with other methylxanthines, is able to modulate both innate and adaptive immunity [24,25]. Therefore, the potential immunomodulatory role of Caff was tested on cultures of MICs. Mel1 and Mel3 CM were analyzed after 0.5, 1, and 2 mM Caff treatment for 72 h. The secretion of 5 out of 12 analyzed cytokines/chemokines was significantly modified, while others were affected to a less significant extent. The effect was dose-dependent; in fact, the most evident variations in the modulation of cytokines/chemokines involved in tumor immunosuppression were detectable at the highest dose of Caff. Namely, interleukin (IL)-1β (IL-1β), interferon-inducible protein 10 (IP-10 or *CXCL10*), macrophage inflammatory protein 1-α (MIP-1α or *CCL3*), and MIP-1β (*CCL4*) levels in CM were significantly reduced after Caff treatment compared to control in both cell lines, whereas regulated and normal T cell expressed and secreted (RANTES or CCL5) levels were reduced only in Mel3 cells (Figure 6).

### 2.3. Network Analysis

**Figure 7:**
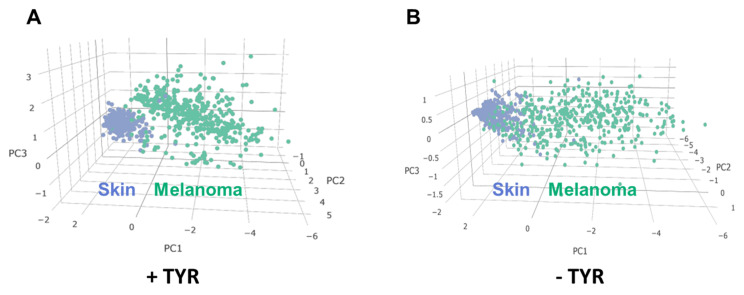
Three-dimensional principal component analysis (PCA) results based on tissue expression values of IL-1β (*IL1B*), IP-10 (*CXCL10*), MIP-1α (*CCL3*), MIP-1β (*CCL4*), and RANTES (*CCL5*) with (**A**) or without (**B**) *TYR*. The two groups (normal skin vs. melanoma) were better spatially discriminated when including TYR. Each point was obtained with specific GEPIA2 graphic tools, with selected data on genes plotted in a three-dimensional space consisting of three principal components (PC1, PC2, and PC3). The blue and green dots represent the normal and melanoma tissues, respectively.

To integrate in silico and in vitro experiments, the aforementioned cytokines/chemokines (IL-1β, IP-10, MIP-1α, MIP-1β, and RANTES) and TYR expression were further analyzed. The combined expression of TYR with cytokines/chemokines secretion modulated in MICs was subject to PCA analysis carried out by the “Dimensionality reduction” tool in GEPIA2. Figure 7A shows PCA analysis of cytokines/chemokines including TYR. It can be observed that the three most relevant components are able to distinguish and cluster melanoma from control cases with high efficacy. On the contrary, PCA analysis performed using the same dataset but in the absence of TYR (Figure 7B) shows a lower ability to discriminate the two groups according to a three-dimensional spatial distribution, confirming a crucial role of TYR in immunomodulatory signals secretion by human MICs upon Caff treatment.

**Figure 8:**
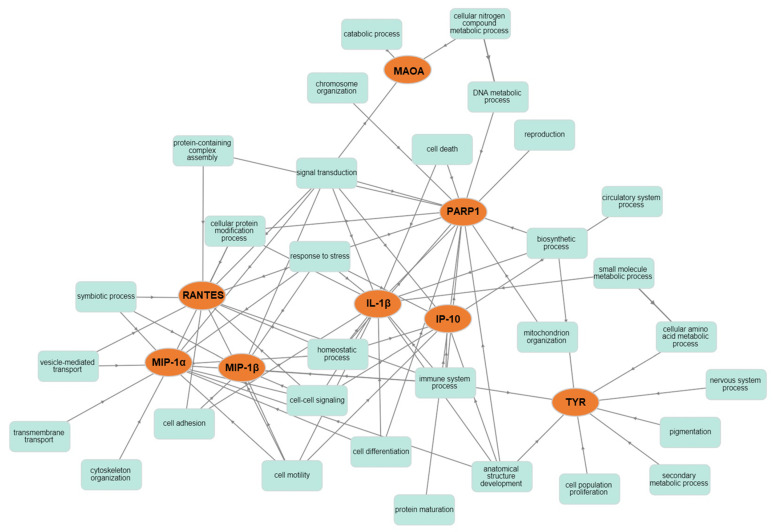
Gene ontology (GO) analysis of molecules derived from the above-mentioned analyses using the GOnet Internet application. Classification of specific proteins was performed according to molecular functions. Arrows indicate direct links between molecular functions according to the GOnet annotation. Round nodes represent genes/proteins with a central function; square nodes represent various molecular functions as identified by the GO analysis.

Finally, all previously identified CMT (MAOA, PARP1 and TYR) and Caff-modulated cytokines/chemokines were uploaded to the online software GOnet. Interestingly, we found that all studied Caff targets were enriched in several biological processes (Figure 8), suggesting not only an important immune-regulative activity of Caff, but also possible effects on differentiation, cytoskeleton reorganization, and cell–cell interactions.

## 3. Discussion

Several studies demonstrate that coffee induces beneficial effects on many human diseases, including cardiovascular, neurodegenerative and cancerous ones [1,2,3,26]. Unfortunately, due to the many roasting and brewing methods for making coffee and coffee-based beverages, the composition of bio-compounds may differ significantly [27]. This makes it very difficult to discriminate and characterize the pharmacological effects due to the numerous bioactive compounds present in coffee [28]. Caff is one of the bioactive molecules found in most coffees and other beverages that are largely used worldwide, and it has been the subject of extensive investigations to characterize its pharmacological effects, such as neuroprotective action [29] or its role in metabolism [30]. Several studies have reported the anti-neoplastic effects of coffee consumption, for instance, by reducing the risk of hepato-carcinoma and breast cancer in post-menopausal women [26]. On the other hand, the studies aimed at identifying the role of single bioactive compounds present in coffee are not yet conclusive. In fact, in caffeine-containing beverages, such as coffee, cocoa, and green and black tea, there is a large variety of other bioactive compounds belonging to polyphenols family [31]. It is well-established that in aqueous solution, Caff is able to interact with polyphenols and other aromatic molecules (e.g., theaflavin, quercetin, epicatechin gallate, and gallic acid) forming crystallin complexes [32,33,34]. Therefore, the bioavailability of Caff depends also on the presence of these complexes [35]. This may explain, at least in part, the discrepancies among published reports focused on such biocomplexes, whose biological effects still represent a very interesting field of research. However, a protective effect of Caff on colorectal cancer has been reported and the molecular mechanisms and targets have been investigated [36]. 

A potentially beneficial effect of Caff on melanoma cells, a highly malignant skin cancer, has been reported in several investigations [37,38]. Nevertheless, the molecular mechanisms and targets have not yet been investigated. Since differentiation and immune response play a crucial role in melanoma development and progression, our study focused on studying the differentiation pathways and immune secretory signals elicited by treatment of human melanoma stem cells with Caff. An in silico screening of 12 Caffeine Molecular Targets (CMT) obtained from published reports on Pubmed indicated eight genes, namely *DNA2*, *MAPK1*, *PTK2B*, *ADORA2A*, *MAOA*, *PARP1*, *TYR*, and *HDAC1*, whose expression was significantly changed in melanoma specimens compared to nevi, and with highly interesting AUC. When these data have been validated on other databases, allowing the evaluation of larger numbers of patients, only *PARP1* and *TYR* have been confirmed as the most significantly related, among the CMT, to severity and survival. Moreover, low *TYR* expression in BRAF-mutated melanoma subtypes showed a significant association with longer survival. Interestingly, a role of brain tyrosinase has been involved in age-dependent neuromelanin production in the pathogenesis of Parkinson’s disease [39], a neurodegenerative disease in which a neuroprotective role of Caff has been reported [29].

These observations prompted us to investigate in depth the role of TYR, the key enzyme in melanin synthesis, in two human melanoma-initiating cell lines, namely Mel1 and Mel3, previously characterized as suitable melanoma cell models for experimentation [22,40]. Both MICs used in the present study have been reported as melanogenetic cell lines, both carrying a V600E BRAF mutation [40]. MICs represents a very useful cancer cell model. In fact, CSCs are also implicated in metastatic dissemination and resistance to therapies; they can be considered directly related to patient outcome [41]. Moreover, CSCs are a subset of cells that are able to self-renew, differentiate into all cell types found in the original tumor, and initiate tumor growth when transplanted into immunocompromised mice [42]. Consequently, since they can be considered a dedifferentiated subpopulation, CSCs are a promising tool for the differentiation therapy [43]. Caff was able to inhibit MICs’ proliferation and spheroid formation in a dose-dependent manner, as well as their melanin production (a melanoma differentiation marker), confirming the in silico data and the hypothesis that one of the key players of Caff’s action on melanoma cells is TYR. The intracellular increase in melanin after Caff exposure has been reported in murine B16-F10 melanoma cells [37,38] and in human MICs in the present study (Figure 5E). However, the effects of methylxanthines on melanogenesis require further investigation. Indeed, it is important to note that some literature data report that Caff is a TYR inhibitor [44,45], but this discrepancy might be due to different Caff concentrations, solvent/salt presence and solubility issues used for in vitro experiments, as described [46]. As previously stated, and as demonstrated by our network analysis (Figure 8), Caff is capable of modulating a large variety of molecular targets. For example, methylxanthines can induce MITF (microphthalmia-associated transcription factor) up-regulation through a cAMP-dependent pathway, resulting in the stimulation of melanogenesis [47]. It is noteworthy that the role of TYR in melanoma remains complex, reflecting the crucial question about the cause/effect relationship of TYR (and melanin) expression. It is well accepted that TYR expression increases during tumorigenesis and represents a specific melanoma-related antigen [48]. In fact, the detection of *TYR* mRNA in peripheral blood relates to the occurrence of melanoma metastases [49]. Nevertheless, amelanotic melanomas show more aggressive features (due also to a higher probability of misdiagnosis) and have a poorer survival rate [50]. This observation is in line with our in silico analyses, which emphasize how low *TYR* levels are associated with poor clinical outcome. Therefore, the use of molecules that have melanin-inducing effects, such as Caff, appears to be an interesting and intriguing field of investigation.

Taking advantage of the MICs model, the effects of Caff were studied to evaluate the secretion of 12 cytokine/chemokines known as important immunoregulatory signals, to understand how cancer stem cells can communicate with the TME. The case of cutaneous melanoma is very interesting as it is considered one of the human cancers whose growth and progression can be positively affected by immunotherapy, which, on the other hand, requires novel approaches aimed at minimizing immune-related toxicity [51], such as, for instance, those based on dietary approaches. We demonstrated that MICs exposed to increasing concentrations of Caff showed a significant reduction in the secretion of IL-1β, IP-10, MIP-1α, MIP-1β, and RANTES. The results obtained through the in silico screening of CMT, i.e., the crucial role of TYR, were combined with those achieved by the in vitro experiments, i.e., indicating an important role of secretory signals such as IL-1β, IP-10, MIP-1α, MIP-1β, and RANTES in MICs exposed to Caff. These findings were subjected to PCA analysis carried out by the “Dimensionality reduction” tool in GEPIA2. The data, shown in Figure 7, indicated that TYR is an essential player in the modulation of the immune response of MICs exposed to Caff. It is worth noting the ability of Caff to modulate the immune response that is achieved by suppressing the secretion of a few cytokines as well as by the reduction in antibody production [24]. A similar immune regulation by Caff has been reported in studies on colorectal cancer [44]. The results of our studies are in line with these findings, indicating a significant effect of Caff in reducing the inflammatory and chemotactic signals secreted by MICs. In the literature, some potent anti-inflammatory actions of melanins have been reported [52], while other immunomodulatory effects of new melanins have been reported in hypoimmune mice [53]; in addition, it has been suggested that the main function of melanocytes and melanin might be immunomodulation [54]. Therefore, the increased melanin production achieved by the involvement of the key biosynthetic enzyme, TYR, might be another weapon to modulate or interfere with the immune system. On the other hand, the role played by inflammation factors in melanogenesis has been studied in depth and reviewed [55], suggesting that inflammation and melanin production may be two interrelated phenomena. Interestingly, some cytokines measured in the present study and also included in CMT (i.e., IL-10, IL-1ra, and TNF-α), as well as INF-γ, are listed among those involved in the control of melanogenesis [55]. Their secretion after Caff treatment was reduced in our experimental settings, although not significantly, and IL-10, IL-1ra, and TNF-α were not validated by GEO/ROC analysis. The slightly different effects of Caff on cytokines secretion by the two MICs models were not surprising, likely reflecting the reported differences in sensitivity to chemotherapeutic agents of patient-derived Mel1 and Mel3 cells in melanospheres viability experiments [40]. The role of TYR on immune regulation has also been supported by the network analysis carried out using the GOnet online resource, where all the CMT and modulated secretory signals appear to be tightly interconnected, with a central role played by immune system processes. Finally, a crucial role of the gut microbiota in the immunomodulation of melanoma patients has recently been demonstrated, with important implications for cancer treatment [56,57]. This opens up a further fascinating point of view for correlating the in vivo effects of Caff, whose modulation of gut microbiota has been reported [58,59], possibly by acting on TYR and melanin production.

## 4. Materials and Methods

### 4.1. Materials

Caffeine (Caff), bovine serum albumin (BSA), buffer saline (PBS), glutamine, synthetic melanin, penicillin (10,000 UI/mL) and streptomycin (10 mg/mL) and all other reagents were obtained from Sigma-Aldrich (St. Louis, MO, USA).

### 4.2. Identification of Caffeine Molecular Targets (CMT) Through Bioinformatic Analyses

Caffeine Molecular Targets (CMT) were derived from the literature [37,60,61,62]. Expression of CMT genes was evaluated in the melanoma GDS1375 dataset, from the GEO database. This human dataset (https://www.ncbi.nlm.nih.gov/sites/GDSbrowser?acc=GDS1375 (accessed on 10 October 2020)) reports the expression profiles of 45 primary malignant melanoma and 18 benign skin nevi, and normal skin samples [63]. 

Genes selected from the CMT, as the most relevant, were subjected to GEO analysis and those having AUC ≥ 0.85 were further studied. The validation of the expression levels of selected genes were verified by two independent databases and online resources, IST Online (http://ist.medisapiens.com/) (accessed on 15 October 2020) and GEPIA2 (http://gepia2.cancer-pku.cn/#index) (accessed on 20 March 2021). These databases are useful for expression profiles of different genes in both cancer and normal tissues. The scatter plots were obtained from IST Online database according to a previously reported procedure [63,64]. Expression analysis, survival mapping, and multiple gene comparison were carried out by the specific tools available at the GEPIA2 database [65]. In particular, the overall survival (OS) method was used for generating heat map analysis in multiple cancer types. High and low cohorts were separated by median. The Kaplan–Meier plots also showed OS and the median was selected as the threshold for separating the high- and low-expression groups (cutoff = 50%). The hazard ratio (HR) was calculated based on the Cox PH Model.

### 4.3. Cell Culture and Proliferation Studies

Two patient-derived BRAF-mutated human melanoma-initiating cell lines (MICs), namely Mel1 and Mel3, were isolated as melanospheres and cultured in a suitable medium, as described previously [40]. For proliferation studies, Caff was dissolved in PBS without Ca^++^/Mg^++^. Mel1 and Mel3 cells were seeded in 12-well ultralow attachment culture plates (BD Falcon, Franklin Lakes, NJ, USA) in complete culture medium, and treated with 1 mM and 2 mM Caff for 72 h. Cells were harvested and counted with a Neubauer modified chamber, after Trypan Blue staining for cytotoxicity evaluation. Long-term effects of Caff on proliferation were established by spheroid formation assay, as described previously [66]. Briefly, MICs were plated (1 × 10^3^ cells/mL) and exposed to Caff for a week. Spheroids were photographed (Olympus BH-2 LH50A) and their number and size (percentage area covered by spheroids) were quantified with ImageJ^®^ (v. 1.5d) software (Wayne Rasband, National Institutes of Health, Bethesda, MD, USA). Data are expressed as a percentage of the control (100%) [67].

### 4.4. Evaluation of Melanin Content

For melanin evaluation, Mel1 and Mel3 cells, treated or not with Caff for 72 h, were washed twice in PBS, collected by centrifugation and counted. Melanin was extracted from cells with 1 mL of 1 M NaOH at 75 °C for 1 h. Pigment content was determined by absorption at 475 nm and compared with the standard curve for synthetic melanin.

### 4.5. Cytokines/Chemokines Profile in Melanospheres Supernatants

Conditioned media (CM) of Mel1 and Mel3 cells untreated and treated with Caff, were collected and centrifuged to remove cell debris. CM were analyzed with a magnetic-bead-based multiplex immunoassay, i.e., xMAP technology on the Luminex platform, carried out according to the manufacturer’s guidelines (Bio-Plex, Bio-Rad Laboratories, Milan, Italy). Cytokines/chemokines were measured with a custom Bio-Rad Bio-Plex human cytokine reagent kit for IP-10, monocyte chemoattractant protein-1, MIP-1α, MIP-1β, RANTES, IL-1β, IL-10, IL-12, IL-17, IL-1ra, TNF-α, and IFN-γ. Data were acquired on the Bio-Rad Bio-Plex X200 reader equipped with Bio-Plex Pro™ Wash Station and analyzed using Bio-Plex Software Manager™ 6.1 (Bio-Rad, Hercules, CA, USA).

### 4.6. Network Analysis

Dimensionality reduction by PCA analysis was carried out by the specific tool available at the GEPIA2 database [65].

Gene Ontology (GO) analysis (significant q-value threshold level of <0.05) of selected caffeine targets was obtained using the GOnet database (https://tools.dice-database.org/GOnet/ (accessed on 20 January 2021)) [68]. Proteins were categorized according to GO (GO term annotation) biological processes.

### 4.7. Statistical Analysis

Results from in vitro experiments are expressed as means of three independent experiments ± standard deviation (SD). The statistical significance of differences was determined by Student’s *t*-test, the significance threshold was set at *p* ≤ 0.05. GraphPad PRISM 5 software (GraphPad Prism Software Inc., San Diego, CA) was sued. Generation of ROC curves from expression data reported in the GDS1375 dataset and calculation of AUC data were performed as previously described [64,69].

## 5. Conclusions

In silico screening of genes potentially involved in the effects of Caff, validated by in vitro analysis with two MICs models exposed to caffeine to evaluate their behaviors, confirms a key role of TYR and melanin, possibly acting through an immunomodulatory action, on melanoma growth and evolution. This opens new viewpoints to explain the powerful antineoplastic effects of caffeine. 

## Figures and Tables

**Table 1:** ROC analysis of CMT, according to the GDS1375 dataset from GEO database. Genes showing expression level fold change higher than 1.4 or lower than 0.6 in melanoma vs. nevi.

Symbol	Mean Expression in Melanoma	Mean Expression in Nevi	*t*-Test (*p* Value)	Fold Change (Melanoma/Nevi)	AUC (ROC Value)	*p* Value of AUC
***DNA2***	**149.7**	**75.49**	**2.22 × 10^−6^**	**1.98**	**0.89**	**<0.0001**
*IL1RN* (IL-1ra)	440.1	827.11	9.00 × 10^−5^	0.53	0.80	0.0001
*SCL2A1* (GLUT-1)	1213.99	1985.62	1.00 × 10^−4^	0.61	0.80	0.0001
***MAPK1***	**733.11**	**1361.07**	**2.30 × 10^−6^**	**0.53**	**0.87**	**0.0001**
***PTK2B***	**1411.28**	**784.15**	**4.40 × 10^−13^**	**1.8**	**0.96**	**<0.0001**
*ADORA1*	299.01	201.01	3.70 × 10^−3^	1.5	0.75	0.001
***ADORA2B***	**217.84**	**564.00**	**2.06 × 10^−11^**	**0.39**	**0.94**	**0.0001**
***MAOA***	**46.83**	**291.86**	**2.48 × 10^−11^**	**0.16**	**0.95**	**0.0001**
*PDE2A*	212.43	427.64	2.10 × 10^−4^	0.49	0.78	0.0004
***PARP1***	**2182.16**	**978.73**	**1.20 × 10^−11^**	**2.23**	**0.98**	**0.0001**
***TYR***	**22881.3**	**7144.98**	**1.25 × 10^−10^**	**3.2**	**0.94**	**0.0001**
***HDAC1***	**1614.36**	**1146.88**	**2.19 × 10^−5^**	**1.41**	**0.86**	**<0.0001**

Bold: ROC curve analyses demonstrated that eight genes can efficiently discriminate melanoma from nevi samples (Area under the curve, AUC > 0.85 and a *p* ≤ 0.0001).

## Data Availability

All data from patients were derived from publicly available databases.

## Supplementary Materials

Table S1: Expression of CMT, according to the GDS1375 dataset from the GEO database. Genes showing expression levels with fold change lower than 1.6 or higher than 0.4 in melanoma vs. nevi. Figure S1: Gene expression comparison of *PARP1* (poly (ADP-ribose) polymerase 1), *MAOA* (monoamine oxidase A), and *TYR* (tyrosinase) in different tumor tissues according to the GEPIA2 database.

## Sample Availability

Not available.
