# Supplementary material for: Targeting Melanoma-Initiating Cells by Caffeine: In Silico and In Vitro Approaches
Source: Molecules. 2021 Jun 13;26(12):3619. doi: 10.3390/molecules26123619 (PMC8231844; doi:10.3390/molecules26123619)
Supplement: Supplementary file 1 [file molecules-26-03619-s001.zip › molecules-1236169-supplementary.pdf]

# Targeting Melanoma-Initiating Cells by Caffeine: In Silico and In Vitro Approaches

**Claudio Tabolacci [1,*], Martina Cordella [1], Stefania Rossi [1], Marialaura Bonaccio [2], Adriana Eramo [1], Carlo Mischiati [3], Simone Beninati [4], Licia Iacoviello [2,5], Antonio Facchiano [6,†] and Francesco Facchiano [1,†]**

[1] Department of Oncology and Molecular Medicine, Istituto Superiore di Sanità, Viale Regina Elena 299, 00161 Rome, Italy; martina.cord87@gmail.com (M.C.); stefania.rossi@iss.it (S.R.); adriana.eramo@iss.it (A.E.); francesco.facchiano@iss.it (F.F.)
[2] Department of Epidemiology and Prevention, IRCCS Neuromed, 86077 Pozzilli, Italy; marialaura.bonaccio@moli-sani.org (M.B.); licia.iacoviello@moli-sani.org (L.I.)
[3] Department of Neuroscience and Rehabilitation, School of Medicine, University of Ferrara, 44121 Ferrara, Italy; msc@unife.it
[4] Department of Biology, University of Rome Tor Vergata, 00133 Rome, Italy; beninati@bio.uniroma2.it
[5] Department of Medicine and Surgery, Research Center in Epidemiology and Preventive Medicine (EPIMED), University of Insubria, 21100 Varese-Como, Italy
[6] Laboratory of Molecular Oncology, IDI-IRCCS, 00167 Rome, Italy; a.facchiano@idi.it
* Correspondence: claudiotabolacci@tiscali.it
† These authors contributed equally to this work.

## Table S1

Expression of CMT, according to the GDS1375 dataset from GEO database. Genes showing expression level with fold change lower than 1.6 or higher than 0.4 in melanoma vs nevi

| Symbol | Mean expression in melanoma | Mean expression in nevi | t-test (*p* value) | Fold change (melanoma/nevi) |
|---|---|---|---|---|
| *ATM* | 456.5 | 448.7 | 0.857 | 1.02 |
| *IL10* | 105.6 | 118.3 | 0.459 | 0.89 |
| *IGF1R* | 942.2 | 1346.6 | 0.012 | 0.69 |
| *EIF2A* | 1898.8 | 1378.2 | $5.03 \times 10^{-6}$ | 1.38 |
| *CASP3* | 342.5 | 388.3 | 0.019 | 0.88 |
| *BCL2* | 1041.6 | 884.3 | 0.113 | 1.18 |
| *MMP2* | 3179.8 | 3539.2 | 0.692 | 0.89 |
| *MMP9* | 2922.7 | 1018.5 | 0.329 | 2.87 |
| *PXN* | 1409.3 | 1112.8 | 0.0005 | 1.27 |
| *ADORA3* | 324.1 | 274.1 | 0.0614 | 1.18 |
| *ACHE* | 167.1 | 119.2 | 0.0501 | 1.41 |
| *TNFA* | 147.3 | 198.4 | 0.044 | 0.74 |
| *TGM2* | 389.8 | 267.5 | 0.0984 | 1.46 |
| *RBBP8* (*SAE2*) | 1084.5 | 980.4 | 0.2311 | 1.11 |

*ATM*, ATM serine/threonine kinase; *IL10*, interleukin 10; *IGF1R*, insulin like growth factor 1 receptor; *EIF2A*, Eukaryotic Translation Initiation Factor 2A; *CASP3*, caspase 3; *BCL2*, BCL2 apoptosis regulator; *MMP*, matrix metalloproteinases; *PXN*, paxillin; *ADORA3*, adenosine receptor 3; *ACHE*, acetylcholinesterase; *TNFA*, tumor necrosis factor alpha; *TGM2*, transglutaminase 2; *RBBP8*, RB binding protein 8, endonuclease.

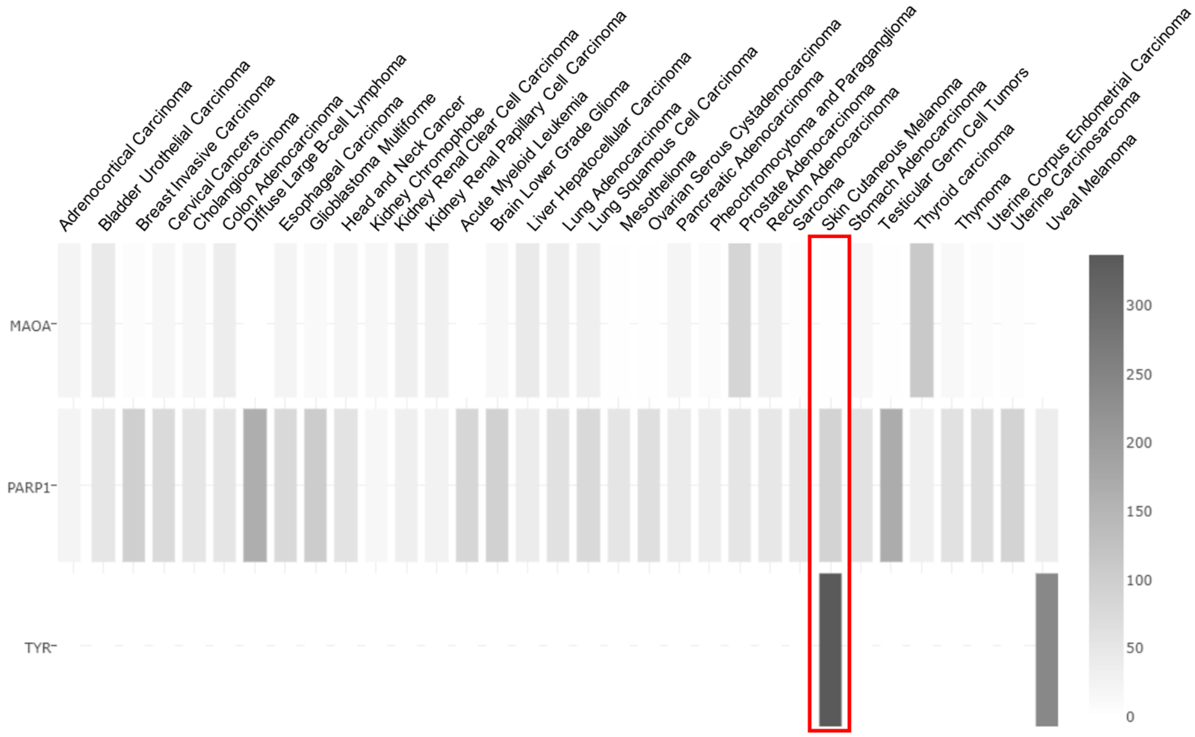


**Figure S1.** Gene expression comparison of *PARP1* (poly(ADP-ribose) polymerase 1), *MAOA* (monoamine oxidase A), *TYR* (tyrosinase) in different tumor tissues according to GEPIA2 database. Red box indicates the skin coutaneous melanoma.
